# Comparison of infant and young child feeding practices and demographic characteristics between healthy and malnourished children aged 0-23 months in Pakistan

**DOI:** 10.12669/pjms.41.2.10243

**Published:** 2025-02

**Authors:** Saba Shahid, Shaima Hamid, Nida Ghouri, Shanila Memon, Rafia Jabbar

**Affiliations:** 1 Saba Shahid, MBBS, MCPS, FCPS Pediatric Consultant, Department of Pediatrics, The Indus Hospital, Karachi, Pakistan; 2 Shaima Hamid, MBBS, FCPS scholar Resident Medical Officer, The Indus Hospital, Karachi, Pakistan; 3 Nida Ghouri Senior Research Scientist, Research Department, The Indus Hospital, Karachi, Pakistan; 4 Shanila Memon Senior Registrar, The Indus Hospital, Karachi, Pakistan; 5 Rafia Jabbar, MBBS, FCPS scholar Resident Medical Officer, The Indus Hospital, Karachi, Pakistan

**Keywords:** Healthy, Infant & young child feeding, Malnourished, Socioeconomic

## Abstract

**Background and Objective:**

Children living in low and middle-income class settings exhibit varied nutritional status ranging from healthy to malnourished. Differences in socioeconomic conditions and Infant & young child feeding practices may be responsible for nutritional disparities among them. This case-control study aimed to explore these differences among healthy and malnourished children aged 0-23 months.

**Methods:**

This was a hospital-based, case-control study. The study was conducted in the outpatient department of The Indus Hospital, Karachi from June 2022 till December 2022. A total of 380 children aged 0-23 months were recruited from the outpatient department.. Compliance to different IYCF indicators, ranging from breastfeeding to complementary feeding, was. assessed by taking 24 hours food recall. Mid-upper arm circumference was used to categorize the nutritional status of children.

**Results:**

The dominant food groups consumed were roots, grains, and tubers, while consumption of vitamin A-rich fruits, flesh food, eggs, fruits, and vegetables were relatively low in both groups. Multivariate logistic regression analysis revealed that low paternal education (COR = 3.64; 95% CI: 2.07, 6.40), employment of mother (COR = 2.27; 95% CI: 1.19, 4.36), and achieving minimal meal frequency (AOR = 2.41; 95% CI: 1.09, 5.31) had a negative association with the nutritional status of children while male gender (COR = 0.52; 95% CI: 0.35, 0.79) and maternal education (AOR = 0.20; 95% CI: 0.06, 0.60) and paternal employment (COR = 0.29; 95% CI: 0.14, 0.59) were positively associated with nutritional status.

**Conclusion:**

We conclude that lack of parental education, father unemployment, maternal employment, and minimal meal frequency were significantly associated with malnutrition in children.

## INTRODUCTION

Malnutrition is a major public health issue worldwide, especially affecting children under five years of age, which is the most critical stage of child development.^1^ It contributes to about one-half of all childhood deaths, globally.^1^ WHO and UNICEF estimate that mortality due to malnutrition is highest in South Asian and African countries.^2^ Similarly, in Pakistan, the under-five mortality rate is 61 deaths per 1,000 live births,^3^ Exclusive and optimal breastfeeding in the first six months, followed by age-appropriate complementary feeding practices are vital in preventing malnutrition in children, especially under two years of age.^4^ UNICEF reported that the odds of infants under six months of age being malnourished were 1.7 times higher when exposed to mixed feeding compared to exclusive breastfeeding.^5^ UNICEF has issued feeding recommendations for children under two years of age, called Infant and Young Child Feeding (IYCF) guidelines^4^, which outline eight core-diet indicators starting from breastfeeding, and continuing to complementary feeding.

The National Nutrition Survey of Pakistan (NNS), conducted in 2018, reported the compliance of Pakistani children to IYCF guidelines, lowest adherence to IYCF indicators was noted in the province of Sindh,^5^ where 52% of children were exclusively breast-fed, and 17% received appropriate complementary feeding.^5^ Similarly, a survey conducted in 2014 in Thatta and Sajawal districts of Sindh found only 37% exclusive breastfeeding and 8% minimal acceptable diet practices among children.

Indus Hospital (TIH) is a tertiary care hospital located in Korangi district of Karachi, Sindh. Most of the children coming to the hospital belong to low or middle-income class. Despite having similar backgrounds, these children exhibit a varied nutritional status ranging from healthy to severe malnutrition.^6^ Although local studies have reported compliance with few IYCF indicators, comparing these indicators between healthy and malnourished children is not done to the best of our knowledge.

The current case-control study determines differences in demographic characteristics and feeding practices between healthy and malnourished children aged 0-23 months.

## METHODS

This was a hospital-based, case-control study. The study was conducted in the outpatient department of The Indus hospital, Karachi from June till December 2022, all children aged 0-23 months were included. Cases comprised of malnourished children while the control group consisted of children who were well-nourished and had come to the hospital for consultation for illnesses like cough, diarrhea, or fever.

### Ethical Approval:

Consent from the hospital ethical board, IHHN-IRB# IHHN_IRB_01_005, dated May 19, 2022 as well from the caregivers was taken. Children whose caregivers refused to participate and those who had chronic illness or malabsorption based on medical record were excluded.

### Sampling technique and sample size:

We estimated a sample size of N = 150, after considering 48.4% prevalence of exclusive breast-feeding in Pakistan.^5^ Precision of 8 and 95% CI was considered. For each malnourished child identified (case), one well-nourished child (control), who met the inclusion criteria was selected.

### Data collection:

Nutritional status of the children included in the study was categorized by determining mid-upper arm circumference (MUAC) which was measured on left upper arm by using UNICEF tapes. Measurements were taken by trained medical officers. Values were approximated to 0.1 cm. MUAC cutoffs of < 110 mm and <125 mm were used for infants between six weeks and less than six months and for children between 6-59 months.^7^ Data was collected using a structured, pretested questionnaire in the local language (Urdu). The questions were tested for appropriateness of terms by pilot testing. The dietary history was obtained by using the 24 hours food recall technique. To ensure accuracy responses were recorded as “not known” if the caregivers were unsure of the duration of breastfeeding or frequency of meals.

### Variables:

Independent variables consisted of WHO, IYCF indicators.^8^ Children who were put to the breast within the first hour of birth were considered to have practiced early initiation (EI); exclusive breastfeeding (EBF) comprised of infants 0-6 months of age who were given only breast milk. Children 6-23 months of age who received at least five out of eight defined groups routinely in their diet were categorized to have met minimum dietary diversity (MDD). Proportion of breastfed and non-breastfed children 0-23 months of age, who received solid, semi-solid, or soft foods (including milk feeds for non-breastfed children) the minimum number of recommended times were considered to achieve minimal meal frequency (MMF). Proportion of children 6-23 months of age who received both MDD and MMF were considered to have minimal acceptable diet (MAD). Dependent variables included demographics (age, gender, paternal education and occupation) and food groups.

### Data analysis:

Data was analyzed by using SPSS version 26. Descriptive statistics (median and interquartile range) were calculated for continuous variables while frequency and percentage were calculated for categorical variables. Chi-square test was applied for the comparison of demographics, feeding practices and food groups. Binary logistic regression model was applied to assess the association between nutritional status and the different potential risk factors in this study. The strength of association between the different risk factors and nutritional status were reported using crude and adjusted odds ratios with 95% confidence interval. Variables with *p* ≤ 0.05 in the multivariate logistic regression model were considered as associated factors. Adequacy of the model to fit the outcome variable with the predictors was checked using the Hosmer and Lemeshow test for goodness of fit.

## RESULTS

### Baseline socio-demographic characteristics:

A total of 380 children were enrolled in the study. One hundred and ninety-three children were malnourished while 187 children were healthy. Median age of the mothers was 26 years (IQR, 22-30). The median family income was PKR 20,000 (Table-I).

**Table-I:** Distribution of baseline characteristics of study participants.

Demographics		

Variable		n (%)
Age	<6 months	189 (49.7)
	6-12 months	87 (22.9)
	> 12 months	104 (27.4)
Gender	Male	197 (51.8)
	Female	183 (48.2)
Education of Mother	Uneducated	125 (32.9)
	<10 grade	174 (45.8)
	≥10 grade	81 (21.1)
Education of Father (out of 377)	Uneducated	103 (27.1)
	<10 grade	155 (40.8)
	≥10 grade	119 (31.5)
Occupation of Mother	Unemployed	333 (87.6)
	Employed	47 (12.3)
Occupation of Father	Unemployed	45 (11.8)
	Employed	334 (88.2)
Birth Order	Firstborn	108 (32.0)
	Second	90 (26.7)
	Third onwards	139 (41.2)

### Comparison of demographic characteristics between healthy and malnourished children:

Girls were more malnourished, compared to boys (108 vs 75, p<0.002). Similarly, the absence of education either in mother or father was significantly associated with malnutrition in the child (p<0.001). Paternal unemployment (75.6%) and maternal employment (68%)was higher in malnourished group) and was a significant risk factor of malnutrition (P <0.001) (Table-II).

**Table-II:** Comparison of demographic and nutritional status between the two groups.

Variable	Malnourished n(%)	Well-nourished n(%)	P value
***Child Age***			
<6 months	90 (47.6)	99 (52.4)	0.098^¥^
6 - 12 months	53 (60.9)	34 (39.1)	
>12 months	50 (48.1)	54 (51.9)	
***Gender***			
Male	85 (43.1)	112 (56.9)	0.002^¥^*
Female	108 (59.0)	75 (41.0)	
***Maternal education***			
Uneducated	82 (65.6)	43 (34.4)	<0.001^¥^*
< 10th grade	87 (50.0)	87 (50.0)	
≥ 10thgrade	24 (29.6)	57 (70.4)	
***Paternal education***			
Uneducated	74 (71.8)	29 (28.2)	<0.001^¥^*
< 10th grade	69 (44.5)	86 (55.5)	
≥ 10thgrade	49 (41.2)	70 (58.8)	
***Occupational status of mother***			
Employed	32 (68.1)	15 (31.9)	0.011^¥^*
Un-Employed / Housewife	161 (48.3)	172 (51.7)	
***Occupational status of father***			
Employed	159 (47.5)	176 (52.5)	<0.001^¥^*
Unemployed	34 (75.6)	11 (24.4)	
***Family income (out of 308)***			
< 15,000 PKR	13 (44.8)	16 (55.2)	0.177^¥^
>15,000 - 20,000 PKR	88 (59.1)	61 (40.9)	
21,000 - 24,000 PKR	06 (50.0)	06 (50.0)	
>24,000 PKR	55 (46.6)	63 (53.4)	
***Exclusive Breast Feeding (out of 377)***			
Yes	182 (50.7)	177 (49.3)	0.388^¥^
No	11 (61.1)	07 (38.9)	
***Continued breastfeeding at 12-15 months***			
Yes	42 (36.8)	72 (63.2)	0.002*
No	24 (54.5	20 (45.5)	
Not known	127 (57.2)	95 (42.8)	0.991*
***Continued breastfeeding at 20-23 months***	14 (51.9)	13 (48.1)	
Yes	20(51.3)	19(48.7)	
No	159 (50.6)	155 (49.4)	
Not Known			
***Top Feeding***			
Yes	11 (68.8)	05 (31.3)	0.142^¥^
No	182 (50)	182 (50.0)	
***Minimum acceptable diet***			
Yes	27(42.2)	37 (57.8)	0.131*
No	166 (52.5)	150 (47.5)	
***Minimum meal frequency***			
Yes	59 (55.1)	48 (44.9)	0.561*
No	36 (50.7)	35 (49.3)	
Not known	132 (34.7)	70 (18.4)	
***Minimum Dietary diversity***			
Yes	36 (42.4)	49 (57.6)	0.077*
No	157 (53.2)	138 (46.8)	

¥ Chi-Square test

* Statistically significant.

### Comparison of IYCF indicators and food groups:

Continued breastfeeding till 12-15 months was significantly more in healthy children (63% vs 36.8% p 0.002), (Table-II). Initiation of complementary feeding before six months (57% malnourished vs 43% healthy) and after eight months (72% malnourished vs 28% healthy) was more in malnourished children but results were not statistically significant (p-value; 0.202). IYCF practices were almost similar in both groups except for minimum dietary diversity which was higher in healthy group (58% healthy versus 42% malnourished, p= 0.07) and minimum meal frequency which was higher in malnourished group (55% in malnourished vs 50% in healthy, p 0.5) (Table-II).

### Food groups details:

Consumption of milk other than breast milk (3% in malnourished vs 96% in healthy children, p<0.001), and intake of fruits and vegetables (53% in malnourished vs 76% in healthy children, p<0.004), was lower in malnourished children, (Table-III)**.** Consumption of nuts was nil in both groups. Chicken was the most consumed flesh food (93%) followed by beef (24%), fish, (7%), and mutton (5%). Liver consumption was nil in both groups. Carrot was the only vitamin A-rich food which was eaten abundantly (88%).

**Table-III:** Comparison of food groups between well-nourished and malnourished children.

	Food Item	Malnourished n (%)	Well-nourished n (%)	p-Value
Food Group	Bread			
	No	179 (51.6)	168 (48.4)	0.314^¥^
	Yes	14 (42.4)	19 (57.6)	
	Semolina (suji)			
	No	160 (49.8)	161 (50.2)	0.390^¥^
	Yes	33 (55.9)	26 (44)	
	Rice			
	No	152 (52)	140 (47.9)	0.369^¥^
	Yes	41(46.6)	47(53.4)	
	Khichri			
	No	130 (47.6)	143 (52.4)	0.048^¥^
	Yes	63 (58.9)	44 (41.1)	
	potato			
	No	165 (49.8)	165 (50.2)	0.341^¥^
	Yes Milk (other than breast milk)	28 (57.1)	22 (44.8)	
	No	192 (54.4)	161 (45.6)	<0.001^¥^*
	Yes	1 (3.7)	26 (96.3)	
Dairy Products	Yogurt			
	No	172 (51.5)	162 (48.5)	0.457^¥^
	Yes	21 (45.7)	25 (54.3)	
	Butter			
	No	189 (50.4)	186 (49.6)	0.372 ^α^
	Yes	4 (80)	1 (20)	
	Beef			
	No	183 (50.6)	179 (49.4)	0.679^¥^
	Yes	10 (55.6)	8 (44.4)	
Mutton			
Flesh food	No	193 (51.1)	185 (48.9)	0.242 ^α^
	Yes	0	2 (49.2)	
	Chicken			
	No	154 (50.7)	150 (49.3)	0.918^¥^
	Yes	39 (50.8)	37 (48.7)	
	Carrot			
	No	175 (51.5)	165 (48.5)	0.439^¥^
	Yes	18 (45)	22 (550	
Vitamin-A rich food	Papaya			
	No	192 (50.8)	186 (49.2)	>0.99 ^α^
	Yes	1 (50)	1 (50)	
	Egg (out of 173)			
	No	59 (55.7)	47 (44.3)	0.528^¥^
	Yes	34 (50.7)	33 (49.3)	
Other Fruits & Vegetables	No	167 50.8)	162 (49.2)	0.004^¥^*
	Yes	26 (51)	25 (49)	

α Fischer Exact test

¥ Chi-Square test

* Statistically significant.

### Results of multivariate analysis:

Male gender was associated with 48% [COR: 0.52 (0.35-0.79)] risk of having malnutrition. Maternal education below 10^th^grade increased the odds of having malnutrition by 4.5 times in crude model [COR: 4.52 (2.47-8.27)] however the odds decreased to 1.15 times, when adjusted with other variables [AOR: 1.15 (0.51-2.29)]. Maternal education more than 10^th^grade had 80% less risk of having malnutrition in child as per the adjusted model [AOR: 0.20 (0.06-0.60)]. Paternal education below 10^th^grade increased the odds of having malnutrition in the child by 3.6 times [COR: 3.64 (2.07-6.40)]. Presence of employment in the father was associated with 71% decreased risk of having malnutrition [COR: 0.29 (0.14-0.59)]. Achieving minimum meal frequency increased the odds of having malnutrition by 2.4 times [AOR: 2.41(1.09-5.31)] as per the adjusted model, Table-IV.

**Table-IV:** Comparison of IYCF indicators with nutritional status- crude and adjusted odds ratio.

Variable	Crude OR (95%CI)	p-Value	AOR (95%CI)	p-Value
***Child age***				
Age <6 months	Ref		-	-
Age between 6 months-1 year	1.71 (1.02 - 2.87)	0.041	-	-
Age between 1-2 years	1.01 (0.63 - 1.64)	0.940	-	-
***Child Gender***				
Female	Ref		-	-
Male	0.52 (0.35 - 0.79)	0.002*	-	-
***Mother education***				
Un-Educated	Ref		Ref	
Below 10^th^ Grade	4.52 (2.47 - 8.27)	<0.001*	1.15 (0.51-2.29)	0.735
10^th^ or Above	2.37 (1.35 - 4.16)	0.003*	0.20 (0.06-0.60)	0.004*
***Father education***				
Un-Educated	Ref		-	-
Below 10^th^ Grade	3.64 (2.07 - 6.40)	<0.001*	-	-
10^th^ or Above	1.14 (0.70 - 1.85)	0.580	-	-
***Mother Employment Status***				
Unemployed / Housewife	Ref		-	-
Employed	2.27 (1.19 - 4.36)	0.013*	-	-
***Father Employment Status***				
Unemployed	Ref		-	-
Employed	0.29 (0.14 - 0.59)	0.001*	-	-
***IYCF indicators***				
***Breast feeding within 1 hour***			-	-
No	Ref			
Yes	0.76 (0.50 - 1.18)	0.229		
***Exclusive breastfeeding***			-	-
No	Ref		-	-
Yes	0.65 (0.24 - 1.72)	0.391	-	-
***Dietary diversity score <4***			-	-
No	Ref			
Yes	0.64 (0.93 - 1.05)	0.079	-	-
***Minimal meal frequency***			-	-
No	Ref		Ref	
Yes	1.19 (0.65 - 2.18)	0.561	2.41 (1.09 - 5.31)	0.028*
***Minimum acceptable diet***				
No	Ref			
Yes	0.65 (0.38 - 1.13)	0.133	-	-

OR: Odds Ratio AOR: Adjusted Odds Ratio CI: Confidence Interval

* Statistically significant.

## DISCUSSION

In the present study, we examined differences in the demographic conditions and IYCF practices between healthy and malnourished children aged 0 to 23 months. The results of our study provide several noteworthy findings regarding demographic and feeding practices differences between the two groups. Notably, factors such as low parental education, unemployed status in fathers and employment in mothers were associated with the risk of having malnutrition in children. Similar observations have been reported in Pakistan and other countries. Specifically, paternal unemployment, low daily wages with family monthly income between 10,000-20,000 PKR have emerged as important risk factors for undernutrition in Pakistan.^9^

This might be because paternal education increases employment prospects, thereby mitigating food insecurity. Additionally, educated fathers tend to be more involved with nutritional issues and parenting behaviors 10 Our study highlights a significant correlation between maternal education and decreased risk of malnutrition in children. Many studies have determined the impact of maternal education on children’s health and have identified several factors such as insufficient knowledge of the nutritional requirements^11^, decreased awareness of healthy eating habits^12^, and decreased household decision-making autonomy^13^ as maternal factors contributing to malnutrition. Presently, Pakistan’s female literacy rate stands at 43%.^14^ Moreover, women’s empowerment remains notably low as evidenced by the 2018 Demographic Health Survey, which reports that only 44% of women have a say in health-related decisions, and 47% have access to media in Pakistan.^15^ Several barriers such as early marriages, family restrictions on education, and safety concerns associated with leaving homes have been identified as factors contributing to low education and empowerment among mothers^16^ Keeping in mind the importance of these factors we recommend that investments should be made in education and initiatives for skill development for women.

In our study, we observed a reverse correlation between Infant and Young Child Feeding (IYCF) practices and maternal employment. However, findings from existing literature present inconsistent views on the relationship between maternal employment and childcare. On one hand, studies suggest that working mothers may have less time for breastfeeding and childcare and may seek fewer healthcare visits.^13^ Conversely, employment is reported to offer women better opportunities for contributing to household income and food expenses.^17^ Additionally working outside the home provides mothers with increased mobility and opportunities to access information and support from family and friends.^18^ Therefore, the benefits of higher income and reduced time spent at home are often seen as a trade-off between maternal employment and childcare.

We identified numerous similarities in feeding practices among both groups. The predominant dietary staples for most children included starch-based foods such as rice, khichri, and bread, while the consumption of legumes and nuts remained low. Conversely, foods rich in vitamin A, flesh foods, eggs, fruits, and vegetables were the least consumed items across both groups. Decreased consumption of red meat, fruits, and vegetables can lead to the deficiency of vitamin B12, iron, folic acid, and vitamin D^19^ leading to complications like anemia.^20^

In our opinion, multifaceted interventions are needed to mitigate nutrient deficiencies in children. While awareness-raising media campaigns and individual patient counseling can contribute to improving dietary diversity, complementary strategies such as food fortification and the provision of multiple micronutrient supplementation (MMS) are equally indispensable interventions and should be promoted.

In the present study, children who achieved minimal meal frequency had twice the odds of malnutrition. This surprising result could be attributed to the fact that mothers were offering insufficient quantity of food despite meeting the recommended frequency of meals. Notably, a recent study reported that inadequate food intake is a primary contributor to malnutrition in Pakistan.^21^ We feel that while promoting proper meal frequency is essential, it’s equally crucial to monitor the quantity of food served per meal. Future studies are needed to determine the effect of correct portion size and quantity of food on the nutritional status of children.

The findings of the present study emphasize the multifaceted nature of malnutrition factors. Therefore, initiatives should take into account both the socioeconomic determinants and Infant and Young Child Feeding (IYCF) practices. By addressing these factors together interventions can be made more robust.

### Limitation:

We had a limited sample size, and data was collected primarily from urban socioeconomic conditions, which may preclude the generalization of our results to other socioeconomic and rural settings. Most of the dietary information was based on mothers’ recall of their children’s 24-hour food intake, which could create recall bias.

## CONCLUSION

Lack of parental education, father unemployment, maternal employment, and minimal meal frequency were significantly different between malnourished and well-nourished children. Both socioeconomic and dietary factors affected the nutritional status of children in our study. Therefore, we recommend that these multifaceted factors be carefully considered when nutritional interventions and policies are formulated.

### Authors Contributions:

**SS**, **SH** and **RJ:** Conceptualization, Methodology. **SS**, **SM**, **SH** and **RJ:** Formal analysis. Critical review. **NG** and SS: Data extraction and Curation. **SH**, **RJ** and **SM:** Literature Search, Writing-original draft preparation. **SS** and **NG:** Writing-review and editing. **SH**, **RJ** and **NG:** Supervision. Critical review. All authors have read. Agreed to the published version of the manuscript and are responsible for accuracy and integrity of the work.
